# Cascades of Care After Incidental Findings in a US National Survey of Physicians

**DOI:** 10.1001/jamanetworkopen.2019.13325

**Published:** 2019-10-16

**Authors:** Ishani Ganguli, Arabella L. Simpkin, Claire Lupo, Arlene Weissman, Alexander J. Mainor, E. John Orav, Meredith B. Rosenthal, Carrie H. Colla, Thomas D. Sequist

**Affiliations:** 1 Harvard Medical School, Boston, Massachusetts; 2 Division of General Internal Medicine and Primary Care, Department of Medicine, Brigham and Women’s Hospital, Boston, Massachusetts; 3 Division of General Internal Medicine, Department of Medicine, Massachusetts General Hospital, Boston; 4 American College of Physicians, Philadelphia, Pennsylvania; 5 The Dartmouth Institute for Health Policy and Clinical Practice, Geisel School of Medicine at Dartmouth, Lebanon, New Hampshire; 6 Department of Biostatistics, Harvard T.H. Chan School of Public Health, Boston, Massachusetts; 7 Department of Health Policy and Management, Harvard T.H. Chan School of Public Health, Boston, Massachusetts

## Abstract

**Importance:**

Incidental findings on screening and diagnostic tests are common and may prompt cascades of testing and treatment that are of uncertain value. No study to date has examined physician perceptions and experiences of these cascades nationally.

**Objective:**

To estimate the national frequency and consequences of cascades of care after incidental findings using a national survey of US physicians.

**Design, Setting, and Participants:**

Population-based survey study using data from a 44-item cross-sectional, online survey among 991 practicing US internists in a research panel representative of American College of Physicians national membership. The survey was emailed to panel members on January 22, 2019, and analysis was performed from March 11 to May 27, 2019.

**Main Outcomes and Measures:**

Physician report of prior experiences with cascades, features of their most recently experienced cascade, and perception of potential interventions to limit the negative consequences of cascades.

**Results:**

This study achieved a 44.7% response rate (376 completed surveys) and weighted responses to be nationally representative. The mean (SE) age of respondents was 43.4 (0.7) years, and 60.4% of respondents were male. Almost all respondents (99.4%; percentages were weighted) reported experiencing cascades, including cascades with clinically important and intervenable outcomes (90.9%) and cascades with no such outcome (94.4%). Physicians reported cascades caused their patients psychological harm (68.4%), physical harm (15.6%), and financial burden (57.5%) and personally caused the physicians wasted time and effort (69.1%), frustration (52.5%), and anxiety (45.4%). When asked about their most recent cascade, 33.7% of 371 respondents reported the test revealing the incidental finding may not have been clinically appropriate. During this most recent cascade, physicians reported that guidelines for follow-up testing were not followed (8.1%) or did not exist to their knowledge (53.2%). To lessen the negative consequences of cascades, 62.8% of 376 respondents chose accessible guidelines and 44.6% chose decision aids as potential solutions.

**Conclusions and Relevance:**

The survey findings indicate that almost all respondents had experienced cascades after incidental findings that did not lead to clinically meaningful outcomes yet caused harm to patients and themselves. Policy makers and health care leaders should address cascades after incidental findings as part of efforts to improve health care value and reduce physician burnout.

## Key Points

**Question:**

How common are cascades of care after incidental findings, what occurs during the cascades, and what are the physician-reported consequences?

**Findings:**

Among 991 practicing US internists in this nationally representative survey study, most physicians reported that they had experienced cascades after incidental findings that did not lead to clinically important and intervenable outcomes yet caused harm to patients and themselves.

**Meaning:**

This study’s findings suggest that policy makers and health care leaders should address cascades after incidental findings as part of efforts to improve health care value and reduce physician burnout.

## Teaser

This survey study estimates the national frequency and consequences of cascades of care after incidental findings using results from a national survey of US physicians.

## Introduction

Incidental findings on screening and diagnostic tests are common and may trigger cascades of further testing and treatment that are of uncertain value.^1,2,3,4,5,6,7,8,9,10^ By some estimates, up to 52% of radiology and laboratory tests produce incidental findings,^3,5,11,12,13^ and these rates are likely to increase with gains in technology.^5^ In some cases, further evaluation of these findings may reveal a clinically important and intervenable discovery, such as an early-stage cancer first detected on chest radiography that would have caused death if left untreated. More often, subsequent evaluations may find nothing significant, such as an electrocardiogram anomaly triggering a stress test and cardiac catheterization that ultimately shows no cardiovascular disease.^9,10,14^ Such cascades of care come with substantial potential for harms^9,14,15,16^: patients may experience anxiety and additional treatment risks in addition to monetary costs and inconvenience,^1,7,14,17,18^ and physicians may be distressed, conflicted, or burdened by additional work.^14,16,19,20^

Although this phenomenon has been described anecdotally and in specific clinical contexts, no study to our knowledge has examined the national scope of incidental findings or the cascades that may follow. We surveyed US internists to understand physician perceptions and experiences of these cascades at a national level. We aimed to estimate the frequency of cascades of care after incidental findings, identify the contents and characteristics of these cascades, understand their perceived consequences for patients and physicians, and pinpoint ways to minimize any negative consequences.

## Methods

We conducted a web-based national survey of US internists between January 22 and March 3, 2019, in collaboration with the American College of Physicians (ACP), and analysis was performed from March 11 to May 27, 2019. The study followed the American Association for Public Opinion Research (AAPOR) reporting guidelines and was approved by Partners Institutional Review Board, which also waived the need for participant informed consent.

### Study Population

We used the Internal Medicine Insider Research Panel, a nationally representative panel of ACP member physicians. The ACP Research Center used stratified random sampling to create the panel in 2011 and regularly adjusts the panel to represent its membership across multiple demographic characteristics. Panelists agree to participate in approximately 2 projects per month and are rewarded for survey completion with points redeemable for gift cards.

Within this panel, we identified 991 physicians (57.4% of the member panel) who were residents, fellows, internists, or geriatricians practicing outpatient medicine. We further screened for respondents who were active in medicine, specialized in general internal medicine or geriatrics (among attendings), and practiced primarily or entirely in the outpatient setting.

### Survey Instrument and Measures

Using literature review and clinical knowledge, we developed a survey instrument that included items on physicians’ prior experiences with cascades in general, items focused on their most recently experienced cascades (used to describe a sample of cascades), and an item on interventions to limit the negative consequences of cascades (based on the 2013 Presidential Commission on the Study of Bioethical Issues report on incidental findings).^5,15,21^ We defined incidental findings as “actionable results that are unrelated to why one ordered the tests” and described a cascade stemming from such a finding as “additional medical care, such as telephone calls, office visits, further testing, and treatment.”

We included questions on physician characteristics, including age, race/ethnicity, US vs foreign medical training, trainee vs attending status, clinical site, practice setting (urban, suburban, or rural), time in direct patient care, and malpractice history. We used a validated single-item discomfort with uncertainty scale^22^ ranging from 1 to 6, with 6 signifying the greatest discomfort.^21,23^ We also used a validated 6-item cost-consciousness scale.^24,25,26^ For this scale, we reversed items with negative wording to ensure that a higher score meant greater cost-consciousness, then calculated summary scores by summing the responses (6-36, with 36 denoting the most cost-consciousness). We conducted in-person, 30-minute cognitive interviews with 10 physician experts using thinking-out-loud and retrospective probing approaches^27^ between November 30 and December 10, 2018. These responses were then incorporated into the final 44-item cross-sectional, online survey instrument (eAppendix 1 in the Supplement).

We obtained physician sex from the ACP master file. We correlated physicians’ self-reported practice zip codes with US Census regions as well as with area-level education and income using data from the US Census Bureau American Community Survey.

### Survey Administration

We distributed the survey via email to 991 panel members on January 22, 2019, for a 41-day period. We paid a $10 incentive for survey completion and sent 6 reminders to improve the response rate. Respondents were required to answer all questions in the survey, so there were no missing response items.

### Statistical Analysis

The response rate was calculated using the American Association for Public Opinion’s Research Response Rate 3 definition (eAppendix 2 in the Supplement).^28^ To mitigate nonresponse bias, the ACP provided final survey weights to approximate national ACP membership on the basis of variables for which respondents were significantly different from the overall population, including respondent age category and experience level (eTable 1 in the Supplement).^29,30^ For relevant survey responses, we stratified analyses by trainee vs attending physician status (eTable 2 in the Supplement). To assess physician characteristics associated with physician harm, we built a multivariable logistic regression model in which the outcome was report of any physician harm (anxiety, frustration, and wasted time and effort) from a cascade in the past year, and covariates (chosen based on clinical plausibility) included age, sex, US vs foreign medical school training, trainee vs attending status, practice setting, time in direct patient care, presence of prior medical malpractice lawsuit, discomfort with uncertainty, and cost-consciousness. Two-sided *P* < .05 was considered statistically significant.^31^ All analyses used weighting commands and were performed with Stata statistical software, version 14.2 (StataCorp LLC).

## Results

### Response Rate and Physician Characteristics

We obtained 443 completed surveys, of which 67 were deemed ineligible based on screening criteria. Our final sample included 376 completed surveys, for a response rate of 44.7% (eAppendix 2 in the Supplement). We weighted responses to be nationally representative. Before and after weighting, respondents were predominantly male (weighted 60.4%), worked in urban and suburban settings, and practiced medicine at least 75% of their time (Table 1). The weighted mean (SE) age of respondents was 43.4 (0.7) years. Of the almost one-fifth who reported personal experience of a medical malpractice lawsuit, 11.4% had been sued for missed follow-up of an incidental finding. Respondents scored a mean of 3.8 (95% CI, 3.6-3.9) on the discomfort with uncertainty scale (minimum of 1 and maximum of 6) and 24.9 (95% CI, 24.3-25.5) on the cost-consciousness scale (minimum of 6 and maximum of 36).

**Table 1.** Demographic and Practice Characteristics of Respondents Among 376 Completed Surveys^a^

Characteristic	Respondents, No. (%) (N = 376)
Age, weighted mean (SE), y	43.4 (0.7)
Age, y	
≤39	148 (53.2)
40-55	99 (23.2)
≥56	129 (23.7)
Sex^b^	
Male	221 (60.4)
Female	144 (39.6)
Race	
White	212 (51.3)
Asian/Pacific Islander	89 (27.2)
Black, mixed, other	31 (9.1)
Prefer not to answer	44 (12.4)
Hispanic	
Yes	17 (4.9)
No/prefer not to answer	359 (95.1)
Training	
US medical graduate	277 (69.5)
Foreign medical graduate	99 (30.5)
Status	
Resident	93 (40.9)
Fellow	22 (5.8)
Attending	261 (53.3)
Clinical site	
Solo	39 (7.7)
Group private practice	122 (26.2)
Academic medical center	136 (45.2)
Community/government practice	62 (16.9)
Other, including staff model HMO	17 (4.0)
Practice setting	
Urban	172 (49.8)
Suburban	165 (40.3)
Rural	39 (9.9)
Geographic region^c^	
Northeast	94 (25.2)
Midwest	86 (24.0)
South	107 (29.7)
West	83 (21.2)
Area-level education, weighted % (SE) with high school education^d^	88.0 (0.72)
Area-level income^d^	
Median income <200% below 2017 federal poverty level	117 (35.3)
Median income ≥200% above 2017 federal poverty level	241 (64.7)
Time in direct patient care	
<49%	49 (13.6)
50%-74%	52 (15.8)
≥75%	275 (70.7)
Prior medical malpractice lawsuit	
Never	274 (80.3)
Once	57 (11.1)
More than once	45 (8.6)
Discomfort with uncertainty scale score, weighted mean (95% CI)^e^	3.8 (3.6-3.9)
Cost-consciousness scale score, weighted mean (95% CI)^f^	24.9 (24.3-25.5)

Abbreviation: HMO, health maintenance organization.

^a^ Unless otherwise specified, values are unweighted numbers (weighted percentages). All responses are based on the survey with the following exceptions: sex was drawn from American College of Physicians member data; geographic region, area-level education, and area-level income were derived from physician-reported practice zip codes in US Census Bureau American Community Survey data.

^b^ Sex data were not available for 11 physicians in the survey.

^c^ Geographic region data were not available for zip codes reported by 6 physicians in the sample.

^d^ Area-level education and income data were not available for zip codes reported by 18 physicians in the sample.

^e^ The discomfort with uncertainty scale ranged from 1 to 6, with 6 signifying the greatest discomfort.

^f^ To create the cost-consciousness scale, items with negative wording were reversed to ensure that a higher score meant greater cost-consciousness, then summary scores were calculated by summing the responses (6-36, with 36 denoting the most cost-consciousness).

### Experience of Cascades

**Figure 1.**
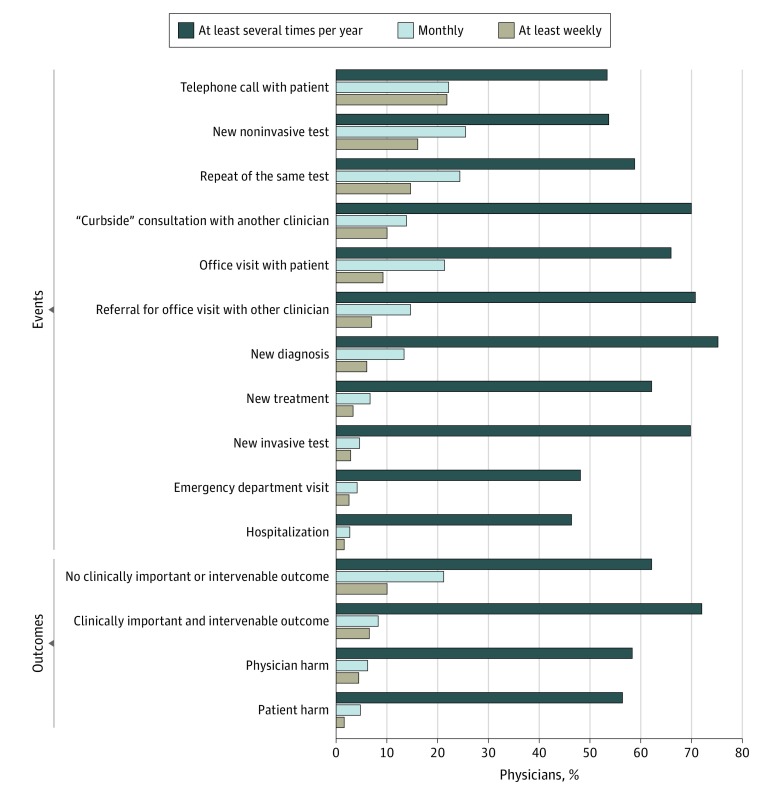
Reported Events and Outcomes of Cascades Following Incidental Findings in the Past Year Values are weighted percentages. There were no missing data. For this analysis, 2 respondents who reported that they experienced no cascades were excluded. The figure shows the distribution of responses to a series of questions: “In the past year, how often did you experience an incidental finding for your patient that led to each of the following?” “Physician harm” included anxiety, frustration, and wasted time and effort. “Patient harm” included physical or psychological harm, treatment burden, disrupted social relationships or status, financial burden, dissatisfaction with care, and death.^15^

Almost all respondents (99.4%) reported that they had experienced cascades resulting from incidental findings as a physician. Many physicians had also experienced cascades when they themselves were the patient (39.3%) or the patient’s family member (54.1%). Physicians reported that cascades for their patients commonly included telephone calls with patients (21.7% reported them at least weekly), new noninvasive tests (16.1% at least weekly), and repeated tests (14.7% at least weekly) (Figure 1). In addition, most physicians had experienced a cascade for their patient that led to a new invasive test (77.2%), emergency department visit (54.8%), or hospitalization (50.6%).

Most physicians (90.9%) had experienced cascades with clinically important and intervenable outcomes, such as discovery of a chronic condition (64.7%), cancer (59.0%), or an acute medical problem (36.6%) as well as cascades with no such outcome (94.4%). When asked about the frequency of experiencing these cascades, physicians were more likely to report that they experienced ones with no clinically important or intervenable outcome (31.1%) than those with meaningful outcomes (14.8%) on at least a monthly basis (Figure 1).

### Perceived Consequences of Cascades

Most physicians reported that cascades had caused their patients harm (86.7%), including psychological harm (68.4%), treatment burden (65.4%), financial burden (57.5%), dissatisfaction with care (27.6%), physical harm (15.6%), disrupted social relationships (8.7%), and death (0.2%). They reported that they personally experienced wasted time and effort (69.1%), frustration (52.5%), and anxiety (45.4%). More than two-thirds (68.9%) of all respondents reported experiencing at least 1 of these harms in the past year. Physicians working in rural areas and those who had greater discomfort with uncertainty were more likely to report experiencing at least 1 of these harms in the past year (Table 2).

**Table 2.** Physician Characteristics Associated With Self-reported Harm From Cascades in the Past Year^a^

Characteristic	% (95% CI)		Adjusted Odds Ratio (95% CI)
	No Harm (n = 112)	Harm (n = 262)	
Age, y			
≤39	31.5 (24.2-39.9)	68.5 (60.1-75.8)	1 [Reference]
40-55	28.4 (19.3-39.6)	71.6 (60.4-80.7)	0.65 (0.26-1.63)
≥56	32.8 (25.2-41.4)	67.2 (58.6-74.8)	0.40 (0.16-1.04)
Sex^b^			
Male	34.8 (28.2-42.2)	65.2 (57.8-71.8)	0.75 (0.43-1.32)
Female	27.2 (20.1-35.7)	72.8 (64.3-79.9)	1 [Reference]
Training			
US medical graduate	27.6 (22.3-33.6)	72.4 (66.4-77.7)	1 [Reference]
Foreign medical graduate	39.0 (28.9-50.2)	61.0 (49.8-71.1)	0.56 (0.31-1.01)
Status			
Trainee	34.8 (26.3-44.4)	65.2 (55.6-73.7)	0.70 (0.28-1.75)
Attending	27.9 (22.7-33.7)	72.1 (66.4-77.3)	1 [Reference]
Practice setting			
Urban	35.6 (28.1-43.9)	64.4 (56.1-71.9)	1 [Reference]
Suburban	30.2 (23.2-38.2)	69.8 (61.8-76.8)	1.28 (0.75-2.20)
Rural	10.9 (4.5-24.3)	89.1 (75.7-95.5)	3.89 (1.38-10.97)^c^
Time in direct patient care			
<49%	28.5 (17.4-43.0)	71.5 (57.0-82.7)	1 [Reference]
50%-74%	38.4 (24.7-54.3)	61.6 (45.8-75.3)	0.91 (0.36-2.34)
≥75%	30.0 (24.4-36.2)	70.1 (63.8-75.6)	1.34 (0.62-2.93)
Prior medical malpractice lawsuit			
Yes	27.3 (19.5-36.8)	72.7 (63.2-80.5)	1.44 (0.75-2.77)
No	32.0 (26.3-38.4)	68.0 (61.6-73.7)	1 [Reference]
Discomfort with uncertainty scale score, mean (95% CI)^d^^,^^e^	3.6 (3.3-3.9)	3.8 (3.7-4.0)	1.23 (1.00-1.50)^c^
Cost-consciousness scale score, mean (95% CI)^d^^,^^f^	24.2 (23.1-25.4)	25.2 (24.6-25.9)	1.05 (1.00-1.10)

^a^ Values are weighted percentages. Data for this analysis were gathered from 374 respondents; 2 respondents who reported that they experienced no cascades were excluded.

^b^ Sex data were not available for 11 physicians in the survey. These physicians were included in the analyses by using an indicator variable for this third category. However, the effect estimates for this category are not presented because of low power and unclear interpretability.

^c^ Statistically significant at *P* < .05.

^d^ The odds ratio represents the increased odds of self-reported physician harm for each additional 1-point increase in discomfort with uncertainty or in cost-consciousness.

^e^ The discomfort with uncertainty scale ranged from 1 to 6, with 6 signifying the greatest discomfort.

^f^ To create the cost-consciousness scale, items with negative wording were reversed to ensure that a higher score meant greater cost-consciousness, then summary scores were calculated by summing the responses (6-36, with 36 denoting the most cost-consciousness).

One-third (33.5%) of physicians reported that their experience of a cascade that uncovered something clinically important and intervenable made them more likely in the future to order the initial test that revealed the incidental finding. Of those who experienced a cascade resulting in no clinically important and intervenable outcome, 31.4% reported that this experience made them less likely to order that same initial test in the future. Finally, of those who experienced a harmful cascade, 30.1% reported that this made them less likely to order that initial test in the future.

### Features of Most Recent Cascades

**Figure 2.**
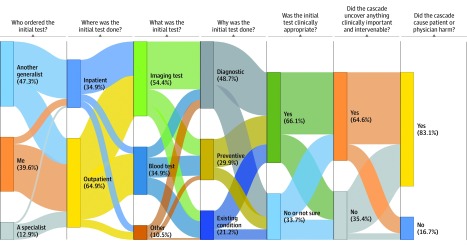
Features of 371 Physicians’ Most Recent Cascades of Care After Incidental Findings Values are weighted percentages. For this analysis, we excluded 2 respondents who reported that they experienced no cascades and 3 respondents for whom it was not possible to assign responses about their most recent cascades to the given categories (eg, the initial event was reported to be a procedure or a panel of unspecified tests). The figure shows the distribution of responses to a series of questions after the following statement: “Thinking back to the last time you experienced any cascade from an incidental finding for your patient….” This “Sankey” diagram shows the percentage of physicians who gave each possible response to questions about their most recently experienced cascade. The heights of the question response boxes are proportional to the percentage of physicians who chose each response (shown in parentheses after the response); the heights of the connecting lines are proportional to the percentage of physicians who then chose the subsequent response. This diagram visualizes the “flow” of the reported cascades from how they started (Who ordered the initial test?) to their outcomes (Did the cascade cause patient or physician harm?). Initial test “Other” includes cardiac, urine, stool, microbiology, and pathology tests.

When asked about their most recent cascade, physicians were most likely to report that the initial test was performed in an outpatient setting (64.9%), was an imaging test (54.4%), and was done for diagnostic purposes (48.7%) (Figure 2). For these results (ie, features of most recent cascades), we restricted our sample to 371 respondents who reported experiencing cascades and for whom their most recent cascades could be assigned to relevant categories. One-third (33.7%) reported that the initial test may not have been clinically appropriate. When faced with the incidental finding, 47.3% reported that they were concerned about an undiagnosed chronic disease, 44.2% were concerned about a new cancer, and 13.0% were concerned about the possibility of an acute event. Of those who pursued further evaluation (unweighted n = 361), the most commonly reported reasons for doing so were because the finding seemed clinically important (59.1%), they were following practice or community norms (43.7%), they were concerned about being sued (30.8%), the patient asked for it (20.1%), or another physician advised it (19.5%) (responses were not mutually exclusive). Most physicians reported that guidelines to inform these evaluations of their most recent cascade either did not exist to their knowledge (53.2%) or were not followed (8.1%). They reported that they attempted to lessen the cascade’s consequences by talking with their patient (62.5%), a specialist (28.9%), or a generalist colleague (18.3%); they also reported consulting educational references (46.8%), guidelines (39.3%), or the primary literature (17.1%). Notably, most cascades that did not uncover anything clinically important or intervenable (86.5%), as well as most cascades that did (81.8%), were reported to cause patient or physician harm (Figure 2).

### Cascade Solutions

Physicians identified several potential options to address these cascades: 62.8% believed that accessible guidelines on how to manage incidental findings would help limit the negative consequences of cascades, 48.1% cited patient and clinician education on potential harms from unnecessary medical care as potentially beneficial, 44.6% identified decision aids (ie, shared decision-making tools), and 42.0% chose malpractice reform. Fewer physicians thought that patient cost-sharing (18.1%) or value-based payment models (16.2%) would help (Table 3).

**Table 3.** Approaches That Respondents Believed Would Help Limit the Negative Consequences of Cascades of Care After Incidental Findings^a^

Approach	% (95% CI)		
	All Respondents (N = 376)	Attending Physicians (n = 261)	Trainee Physicians (n = 115)
Evidence-based recommendations for next steps on radiology and laboratory result reports	66.5 (61.0-71.6)	72.2 (66.4-77.3)	60.0 (50.5-68.9)
Accessible guidelines on how to manage incidental findings	62.8 (57.5-67.9)	61.8 (55.8-67.6)	64.0 (54.6-72.4)
Clinician education on managing incidental findings during training or continuing medical education	54.7 (49.2-60.1)	55.0 (48.9-61.0)	54.4 (44.9-63.5)
Patient and clinician education on potential harms from unnecessary medical care	48.1 (42.7-53.6)	51.7 (45.6-57.8)	44.0 (34.9-53.5)
Shared decision-making tools to aid conversations with patients	44.6 (39.2-50.1)	42.0 (36.0-48.1)	47.6 (38.4-57.1)
Malpractice reform	42.0 (36.8-47.5)	46.8 (40.7-52.9)	36.6 (28.1-46.1)
Patient cost-sharing (ie, insurance plan requires patient to pay a portion of medical costs out of pocket)	18.1 (14.1-22.9)	17.1 (13.0-22.3)	19.1 (12.5-28.0)
Value-based payment models (eg, accountable care organizations)	16.2 (12.1-21.3)	9.4 (6.4-13.7)	23.9 (16.4-33.5)

^a^ Values are weighted percentages. There were no missing data.

### Trainee vs Attending Status

When we stratified our results by trainee vs attending status, we found that attendings generally reported higher frequencies of cascade events than trainees (eFigure in the Supplement). Trainees were more likely than attendings to report consulting a generalist colleague (27.6% vs 9.9%) or reading guidelines (49.2% vs 30.5%) or the primary literature (24.6% vs 10.3%) to shorten or lessen the consequences of their most recent cascade (eTable 2 in the Supplement). Compared with attendings, more trainees believed that value-based payment models would help mitigate cascades (23.9% vs 9.4%), and fewer believed that malpractice reform would (36.6% vs 46.8%, a nonsignificant difference) (Table 3).

## Discussion

In this national survey of US internists, we found that almost all had experienced cascades after incidental findings. We quantified the scope and contents of these cascades. Most physicians reported that incidental findings frequently prompted telephone calls with patients and repeated tests; most physicians had also seen their patients undergo new invasive tests, emergency department visits, and hospitalizations after an incidental finding. Many reported that they had experienced cascades as patients themselves. Physicians reported experiencing cascades for their patients that led to no clinically important or intervenable outcome more often than those that led to meaningful outcomes. Most physicians reported that the cascades had caused psychological or physical harms to patients and to themselves.

This work suggests that cascades are widely prevalent and may seem inevitable—even if not clinically significant—once an incidental finding is discovered.^4,9^ Indeed, the term *cascade* was coined to convey a sequence of events set irrevocably into motion,^8,9^ and a qualitative study^4^ found that primary care physicians reported feeling “compelled but frustrated” to pursue the “quagmire” of costly follow-up evaluations for incidental findings that were unlikely to be significant. Therefore, a key intervention may be to avoid that initial test whenever possible.^9,32^ One-third of physicians in our survey reported that the initial test in their most recently experienced cascade may not have been clinically appropriate: harms are unlikely to be offset by any benefits from testing in such cases.^2,10^

Most physicians reported negative consequences from cascades, including anxiety, frustration, and wasted time and effort. This was particularly true for those physicians reporting higher levels of discomfort with uncertainty, consistent with prior work linking discomfort with uncertainty and measures of burnout and depression.^33^ At a time of great concern about physician burnout and its potential drivers,^34,35^ our findings add to research suggesting that clinician involvement with what they perceive as futile or nonbeneficial care is linked to burnout and its components.^36,37,38^ Professional burnout has numerous negative consequences, with profound implications for physician well-being,^39^ patient satisfaction and safety,^40,41^ and quality of health care delivery.^42,43^ Understanding factors associated with burnout is an important step to enhancing physician well-being, with downstream consequences for high-quality patient care. Physicians who reported negative repercussions from cascades were also more likely to work in rural settings. This may reflect the effect that working in social and supportive environments has on well-being and may suggest that interventions to increase relational connection and community could help.^44^

The frequency of perceived harms from cascades—even from cascades with clinically important and intervenable outcomes—also shows that we need better ways to navigate incidental findings once they are found. Physicians in our survey believed that point-of-care tools might mitigate cascade burden, in line with suggestions by the 2013 Presidential Commission for the Study of Bioethical Issues report on incidental findings.^5^ Specifically, most respondents pointed to evidence-based recommendations written into radiology and laboratory result reports.^4,14,45,46^ Recommendations that quantify the likelihood of various differential diagnoses and are stratified by patient phenotypes (eg, level of anxiety and low vs high disease risk factors), in particular, may allow internists to make more nuanced evidence-based decisions with their patients.^4^ Respondents also cited the need for accessible guidelines on managing incidental findings. Such guidelines are in short supply,^5^ although organizations, such as the American College of Radiology Incidental Findings Committee, are working to fill this gap.^5,14^ Unfortunately, there is no guarantee that guidelines will lead to fewer harms from cascades^47^; indeed, in some cases, guidelines may lead radiologists to recommend, and ordering physicians to pursue, follow-up more often or more intensively than they otherwise would.^48^ Therefore, all of these efforts require more robust cost-effectiveness, decision analysis, and outcome studies of incidental findings to inform thoughtful recommendations and guidelines.^11^

Finally, our results highlight the importance of engaging patients in these efforts (eg, talking to patients about the possibility of incidental findings, even before ordering a test). Recognizing the uncertainty inherent in many of these conversations, this also requires strategies to help physicians with this communication.^49,50^ Almost half of respondents cited patient and clinician education on potential harms from unnecessary medical care as a solution.^5,9^ If (or when) incidental findings arise, clinicians and patients could use decision aids incorporating factors like clinical significance and actionability of the finding, patient time, and cost to decide next steps.^5,6,51,52,53^ Clinicians might also use scripts to help frame an incidental finding and the need for evaluation. Although only 19.9% of physicians reported that they ordered follow-up testing because their patient asked for it, we expect patients to have a bigger role as they gain increased access to laboratory and radiology results through electronic portals. Early, open, and evidence-driven communication with patients may also reassure those physicians who pursue cascades not because they are clinically needed but out of obligation to community norms or fear of getting sued. Almost half of physicians reported that malpractice reform would help reduce cascades, although few believed that payment reforms like patient cost-sharing and value-based payment models would be effective solutions, in keeping with the notion that the decision to pursue cascades is not driven by financial incentives.^54^

### Limitations

This study has several limitations. We cannot determine if physicians would act in practice as they reported in hypothetical scenarios. We achieved a 44.7% response rate, raising the possibility of response bias. However, respondents were found to be different from nonrespondents on just 2 observable demographic characteristics (experience level and age category), by which we weighted our results. We also acknowledge the possibility of recall bias. For example, physicians may better remember emotionally salient events, leading to overestimation of cascades resulting in clinically important and intervenable outcomes or harm and underestimation of cascades resulting in no clinically important or intervenable outcome. Future work might examine the role of the health literacy, patient-clinician relationships, and patient and clinician anxiety.

## Conclusions

The survey findings indicated high national rates of internists experiencing cascades of care after incidental findings that did not lead to clinically important outcomes yet caused harm to patients and physicians. Policy makers and health care leaders can address cascades after incidental findings in their efforts to improve the value of health care and to reduce physician burnout. Initiatives could include the development and effective dissemination of point-of-care guidelines and shared decision-making tools, along with other strategies to embrace and communicate uncertainty.

## Notes

Supplement. **eAppendix 1.** Survey Instrument **eAppendix 2.** Response Rate Calculation **eTable 1.** Sample Weights **eTable 2.** Selected Survey Responses Stratified by Attending vs Trainee Physician Status **eFigure.** Reported Events and Outcomes of Cascades Following an Incidental Finding, Stratified by Attending vs Trainee Physician Status **eReference.**

## References

[1] Rothberg MB A piece of my mind: the $50,000 physical. JAMA. 2014;311(21):-. doi:10.1001/jama.2014.3415 24893085

[2] Bhatia RS, Bouck Z, Ivers NM, Electrocardiograms in low-risk patients undergoing an annual health examination. JAMA Intern Med. 2017;177(9):1326-1333. doi:10.1001/jamainternmed.2017.2649 28692719 PMC5710571

[3] Gundareddy VP, Maruthur NM, Chibungu A, Bollampally P, Landis R, Eid SM Association between radiologic incidental findings and resource utilization in patients admitted with chest pain in an urban medical center. J Hosp Med. 2017;12(5):323-328. doi:10.12788/jhm.2722 28459900

[4] Zafar HM, Bugos EK, Langlotz CP, Frasso R “Chasing a ghost”: factors that influence primary care physicians to follow up on incidental imaging findings. Radiology. 2016;281(2):567-573. doi:10.1148/radiol.2016152188 27192458 PMC5084970

[5] Presidential Commission for the Study of Bioethical Issues Anticipate and communicate: ethical management of incidental and secondary findings in the clinical, research, and direct-to-consumer contexts. https://bioethicsarchive.georgetown.edu/pcsbi/node/3183.html. Accessed September 9, 2019. 10.1093/aje/kwu217 25150271

[6] Westbrook JI, Braithwaite J, McIntosh JH The outcomes for patients with incidental lesions: serendipitous or iatrogenic? AJR Am J Roentgenol. 1998;171(5):1193-1196. doi:10.2214/ajr.171.5.9798845 9798845

[7] Casarella WJ A patient’s viewpoint on a current controversy. Radiology. 2002;224(3):927-927. doi:10.1148/radiol.2243020024 12202734

[8] Mold JW, Stein HF The cascade effect in the clinical care of patients. N Engl J Med. 1986;314(8):512-514. doi:10.1056/NEJM198602203140809 3945278

[9] Deyo RA Cascade effects of medical technology. Annu Rev Public Health. 2002;23:23-44. doi:10.1146/annurev.publhealth.23.092101.134534 11910053

[10] Ganguli I, Lupo C, Mainor AJ, Prevalence and cost of care cascades after low-value preoperative electrocardiogram for cataract surgery in fee-for-service Medicare beneficiaries [published online June 3, 2019]. JAMA Intern Med. doi:10.1001/jamainternmed.2019.1739 31158270 PMC6547245

[11] O’Sullivan JW, Muntinga T, Grigg S, Ioannidis JPA Prevalence and outcomes of incidental imaging findings: umbrella review. BMJ. 2018;361:k2387. doi:10.1136/bmj.k2387 29914908 PMC6283350

[12] Tewari A, Shuaib W, Maddu KK, Incidental findings on bedside ultrasonography: detection rate and accuracy of resident-performed examinations in the acute setting. Can Assoc Radiol J. 2015;66(2):153-157. doi:10.1016/j.carj.2014.04.004 25442905

[13] Grieme CV, Voss DR, Olson KE, Davis SR, Kulhavy J, Krasowski MD Prevalence and clinical utility of “incidental” critical values resulting from critical care laboratory testing. Lab Med. 2016;47(4):338-349. doi:10.1093/labmed/lmw044 27708170

[14] Berland LL, Silverman SG, Gore RM, Managing incidental findings on abdominal CT: white paper of the ACR Incidental Findings Committee. J Am Coll Radiol. 2010;7(10):754-773. doi:10.1016/j.jacr.2010.06.013 20889105

[15] Korenstein D, Chimonas S, Barrow B, Keyhani S, Troy A, Lipitz-Snyderman A Development of a conceptual map of negative consequences for patients of overuse of medical tests and treatments. JAMA Intern Med. 2018;178(10):1401-1407. doi:10.1001/jamainternmed.2018.3573 30105371 PMC7505335

[16] Booth TC, Boyd-Ellison JM The current impact of incidental findings found during neuroimaging on neurologists’ workloads. PLoS One. 2015;10(2):e0118155. doi:10.1371/journal.pone.0118155 25723558 PMC4344225

[17] Rao VM, Levin DC The overuse of diagnostic imaging and the Choosing Wisely initiative. Ann Intern Med. 2012;157(8):574-576. doi:10.7326/0003-4819-157-8-201210160-00535 22928172

[18] Wiener RS, Gould MK, Woloshin S, Schwartz LM, Clark JA What do you mean, a spot? a qualitative analysis of patients’ reactions to discussions with their physicians about pulmonary nodules. Chest. 2013;143(3):672-677. doi:10.1378/chest.12-1095 22814873 PMC3590883

[19] Wiener RS, Schwartz LM, Woloshin S When a test is too good: how CT pulmonary angiograms find pulmonary emboli that do not need to be found. BMJ. 2013;347:f3368. doi:10.1136/bmj.f3368 23820021 PMC4688549

[20] Ofri D Perchance to think. N Engl J Med. 2019;380(13):1197-1199. doi:10.1056/NEJMp1814019 30917257

[21] Tilburt JC, Wynia MK, Sheeler RD, Views of US physicians about controlling health care costs. JAMA. 2013;310(4):380-388. doi:10.1001/jama.2013.8278 23917288 PMC5553287

[22] Gerrity MS, White KP, DeVellis RF, Dittus RS Physicians’ reactions to uncertainty: refining the constructs and scales. Motiv Emot. 1995;19(3):175-191. doi:10.1007/BF02250510

[23] Colla CH, Kinsella EA, Morden NE, Meyers DJ, Rosenthal MB, Sequist TD Physician perceptions of Choosing Wisely and drivers of overuse. Am J Manag Care. 2016;22(5):337-343. 27266435

[24] Schutte T, Tichelaar J, Nanayakkara P, Richir M, van Agtmael M Students and doctors are unaware of the cost of drugs they frequently prescribe. Basic Clin Pharmacol Toxicol. 2017;120(3):278-283. doi:10.1111/bcpt.12678 27639184

[25] Bovier PA, Martin DP, Perneger TV Cost-consciousness among Swiss doctors: a cross-sectional survey. BMC Health Serv Res. 2005;5(72):72. doi:10.1186/1472-6963-5-72 16281977 PMC1308814

[26] Goold SD, Hofer T, Zimmerman M, Hayward RA Measuring physician attitudes toward cost, uncertainty, malpractice, and utilization review. J Gen Intern Med. 1994;9(10):544-549. doi:10.1007/BF02599278 7823224

[27] Willis GB, Artino AR Jr What do our respondents think we’re asking? using cognitive interviewing to improve medical education surveys. J Grad Med Educ. 2013;5(3):353-356. doi:10.4300/JGME-D-13-00154.1 24404294 PMC3771159

[28] Smith TW A revised review of methods to estimate the status of cases with unknown eligibility. https://www.aapor.org/AAPOR_Main/media/MainSiteFiles/FindingE.pdf. Published August 2009. Accessed May 23, 2019.

[29] Davern M Nonresponse rates are a problematic indicator of nonresponse bias in survey research. Health Serv Res. 2013;48(3):905-912. doi:10.1111/1475-6773.12070 23656501 PMC3681235

[30] Pew Research Center. Assessing the representativeness of public opinion surveys. https://www.people-press.org/2012/05/15/assessing-the-representativeness-of-public-opinion-surveys/. Published May 15, 2012. Accessed June 5, 2019.

[31] Althouse AD Adjust for multiple comparisons? it’s not that simple. Ann Thorac Surg. 2016;101(5):1644-1645. doi:10.1016/j.athoracsur.2015.11.024 27106412

[32] Colla CH, Mainor AJ, Hargreaves C, Sequist T, Morden N Interventions aimed at reducing use of low-value health services: a systematic review. Med Care Res Rev. 2017;74(5):507-550. doi:10.1177/1077558716656970 27402662

[33] Simpkin AL, Khan A, West DC, Stress from uncertainty and resilience among depressed and burned out residents: a cross-sectional study. Acad Pediatr. 2018;18(6):698-704. doi:10.1016/j.acap.2018.03.002 29524616

[34] Shanafelt TD, Hasan O, Dyrbye LN, Changes in burnout and satisfaction with work-life balance in physicians and the general US working population between 2011 and 2014. Mayo Clin Proc. 2015;90(12):1600-1613. doi:10.1016/j.mayocp.2015.08.023 26653297

[35] Rotenstein LS, Torre M, Ramos MA, Prevalence of burnout among physicians: a systematic review. JAMA. 2018;320(11):1131-1150. doi:10.1001/jama.2018.12777 30326495 PMC6233645

[36] Chamberlin P, Lambden J, Kozlov E, Clinicians’ perceptions of futile or potentially inappropriate care and associations with avoidant behaviors and burnout. J Palliat Med. 2019;22(9):1039-1045. doi:10.1089/jpm.2018.0385 30874470 PMC6735323

[37] Meltzer LS, Huckabay LM Critical care nurses’ perceptions of futile care and its effect on burnout. Am J Crit Care. 2004;13(3):202-208. 15149054

[38] Schwarzkopf D, Rüddel H, Thomas-Rüddel DO, Perceived nonbeneficial treatment of patients, burnout, and intention to leave the job among ICU nurses and junior and senior physicians. Crit Care Med. 2017;45(3):e265-e273. doi:10.1097/CCM.0000000000002081 27776092

[39] Mateen FJ, Dorji C Health-care worker burnout and the mental health imperative. Lancet. 2009;374(9690):595-597. doi:10.1016/S0140-6736(09)61483-5 19699996

[40] Fahrenkopf AM, Sectish TC, Barger LK, Rates of medication errors among depressed and burnt out residents: prospective cohort study. BMJ. 2008;336(7642):488-491. doi:10.1136/bmj.39469.763218.BE 18258931 PMC2258399

[41] West CP, Tan AD, Habermann TM, Sloan JA, Shanafelt TD Association of resident fatigue and distress with perceived medical errors. JAMA. 2009;302(12):1294-1300. doi:10.1001/jama.2009.1389 19773564

[42] Dyrbye LN, Shanafelt TD Physician burnout: a potential threat to successful health care reform. JAMA. 2011;305(19):2009-2010. doi:10.1001/jama.2011.652 21586718

[43] Dewa CS, Loong D, Bonato S, Thanh NX, Jacobs P How does burnout affect physician productivity? a systematic literature review. BMC Health Serv Res. 2014;14(1):325. doi:10.1186/1472-6963-14-325 25066375 PMC4119057

[44] Simpkin AL, Chang Y, Yu L, Campbell EG, Armstrong K, Walensky RP Assessment of job satisfaction and feeling valued in academic medicine. JAMA Intern Med. 2019;179(7):992-994. doi:10.1001/jamainternmed.2019.0377 31058983 PMC6503555

[45] Elias RM, Sykes AMG, Knudsen JM, Morgenthaler TI Impact of a standardized recommendation and electronic prompts on follow-up of indeterminate pulmonary nodules found on computed tomography. J Pulmonar Respir Med. 2012;2(1). doi:10.4172/2161-105X.1000113

[46] Ware JB, Jha S, Hoang JK, Baker S, Wruble J Effective radiology reporting. J Am Coll Radiol. 2017;14(6):838-839. doi:10.1016/j.jacr.2017.01.045 28434849

[47] Benavidez G, Frakt AB Fixing clinical practice guidelines. https://www.healthaffairs.org/do/10.1377/hblog20190730.874541/full/. Published August 5, 2019. Accessed August 11, 2019.

[48] Cook TS, Zimmerman SL, Jha S Analysis of statistical biases in studies used to formulate guidelines: the case of arrhythmogenic right ventricular cardiomyopathy (ARVC). Acad Radiol. 2015;22(8):1010-1015. doi:10.1016/j.acra.2015.04.009 26100190

[49] Simpkin AL, Armstrong KA Communicating uncertainty: a narrative review and framework for future research [published online June 13, 2019]. J Gen Intern Med. doi:10.1007/s11606-019-04860-8 31197729 PMC6848305

[50] Gheihman G, Johnson M, Simpkin AL Twelve tips for thriving in the face of clinical uncertainty [published online March 26, 2019]. Med Teach. doi:10.1080/0142159X.2019.1579308 30912996

[51] Shickh S, Clausen M, Mighton C, Evaluation of a decision aid for incidental genomic results, the Genomics ADvISER: protocol for a mixed methods randomised controlled trial. BMJ Open. 2018;8(4):e021876. doi:10.1136/bmjopen-2018-021876 29700101 PMC5922516

[52] Reuland DS, Cubillos L, Brenner AT, Harris RP, Minish B, Pignone MP A pre-post study testing a lung cancer screening decision aid in primary care. BMC Med Inform Decis Mak. 2018;18(1):5. doi:10.1186/s12911-018-0582-1 29325548 PMC5765651

[53] Beach MC, Sugarman J Realizing shared decision-making in practice. JAMA. 2019;322(9):811-812. doi:10.1001/jama.2019.9797 31343669 PMC8786261

[54] Ganguli I, Simpkin AL, Colla CH, Why do physicians pursue cascades of care after incidental findings? a national survey [published online July 25, 2019]. J Gen Intern Med. doi:10.1007/s11606-019-05213-1 31346910 PMC7174496

