# Error in Key Points

**DOI:** 10.1001/jamanetworkopen.2019.16768

**Published:** 2019-11-08

In the Original Investigation titled “Cascades of Care After Incidental Findings in a US National Survey of Physicians,”^1^ published October 16, 2019, the Findings portion of the Key Points erroneously stated the number of study participants in the survey sample (991) rather than the number of responding participants (376). This article has been corrected.^1^
