# Supplementary material for: Cascades of Care After Incidental Findings in a US National Survey of Physicians
Source: JAMA Netw Open. 2019 Oct 16;2(10):e1913325. doi: 10.1001/jamanetworkopen.2019.13325 (PMC6806665; doi:10.1001/jamanetworkopen.2019.13325)
Supplement: Supplement. — eAppendix 1. Survey Instrument eAppendix 2. Response Rate Calculation eTable 1. Sample Weights eTable 2. Selected Survey Responses Stratified by Attending vs Trainee Physician Status eFigure. Reported Events and Outcomes of Cascades Following an Incidental Finding, Stratified by Attending vs Trainee Physician Status eReference. [file jamanetwopen-2-e1913325-s001.pdf]

Supplementary Online Content

Ganguli I, Simpkin AL, Lupo C, et al. Cascades of care after incidental findings in a US national survey of physicians. *JAMA Netw Open*. 2019;2(10):e1913325. doi:10.1001/jamanetworkopen.2019.13325

eAppendix 1. Survey Instrument

eAppendix 2. Response Rate Calculation

eTable 1. Sample Weights

eTable 2. Selected Survey Responses Stratified by Attending vs Trainee Physician Status

eFigure. Reported Events and Outcomes of Cascades Following an Incidental Finding, Stratified by Attending vs Trainee Physician Status

eReference.

This supplementary material has been provided by the authors to give readers additional information about their work.

eAppendix 1. Survey Instrument

Clinical Decision Making Survey
January 11, 2019

1. **Which of the following best describes you at this time? (Choose one)**

Resident/Fellow in training	Skip to Q.4
Completed training and active in medicine	
Fully retired/currently not working in medicine	Terminate

2. **Which of the following best describes the location of your patient care?**

No patient care	Terminate
All outpatient	
Primarily outpatient with some inpatient	
Primarily inpatient with some outpatient	Terminate
All inpatient	Terminate

3. **What is your <u>primary</u> specialty area (i.e., the specialty area where you spend most of your time)? (Choose one)**

General Internal Medicine	
Geriatric medicine	
Other	Terminate

4. **Your patient is a healthy 60-year-old man with no prior history of smoking or cancer. Before knee surgery, he receives a routine pre-operative chest x-ray that shows a pulmonary nodule. The radiology report for the follow-up chest computed tomography (CT) scan confirms a 5mm nodule and instructs you to "repeat chest CT in 6-12 months." The 2017 Fleischner Society guidelines recommend no further action. In addition to speaking with your patient, what would you do next?**

Repeat chest CT in 6 – 12 months
Refer patient for consultation with pulmonology or thoracic surgery
Make a decision after speaking with a radiologist, pulmonologist, or thoracic surgeon
No further action per guidelines

5. Your patient is a healthy 30-year-old woman. Before knee surgery, she receives a routine pre-operative complete blood count that is normal except for Hemoglobin 11.2 g/dL (Reference range 11.5 - 16.4 g/dL) and Hematocrit 35.0 (Reference range 36.0 – 48.0%). In addition to speaking with your patient, what would you do next?

Repeat complete blood count in 1 – 4 weeks
Order further tests such as stool guaiac, iron studies, vitamin B12, folate, reticulocyte count, or peripheral smear
Refer to hematology
No further action (result may be due to menstrual blood loss, for example)

6. It is common for screening and diagnostic tests to show incidental findings (i.e., actionable results that are unrelated to why one ordered the tests). These findings may prompt "cascades" of additional medical care such as telephone calls, office visits, further testing, and treatment. Have you ever experienced an incidental finding for your patient that led to a cascade?

Yes
No Skip to Q. 32

7. In the past year, how often did you experience an incidental finding for your patient that led to each of the following?

	**Never**	**Once**	**Several times/year**	**Monthly**	**Weekly**	**Several times/week**	**Daily**
Phone call with patient							
Office visit with patient							
Informal/curbside conversation with specialist or other clinician							
Referral for office visit with other clinician							
Repeat of the same test							
New non-invasive test (e.g., blood test)							
New invasive test (e.g., biopsy)							
New treatment							
Emergency department visit							
Hospitalization							

New diagnosis whether or not clinically important							

8. A "cascade" may uncover something clinically important and intervenable. If you have ever experienced a cascade following an incidental finding that uncovered something clinically important and intervenable, what did the cascade(s) uncover? (Choose all that apply)

Acute medical problem (e.g., heart attack, pulmonary embolism, hyperkalemia)
Chronic disease or condition (e.g., chronic kidney disease, uterine fibroids)
Malignancy
Other (Please Describe: ______________________________)
I have never experienced a cascade that uncovered something clinically important and intervenable (Exclusive) Skip to Q. 10

9. In the past year, how often did you experience a "cascade" following an incidental finding that uncovered something clinically important and intervenable?

Daily
Several times a week
Weekly
Monthly
Several times in the year
Once
Never in the past year

10. Have you ever experienced a "cascade" following an incidental finding for your patient that resulted in NO clinically important or intervenable outcome?

Yes
No Skip to Q. 12

11. In the past year, how often did you experience a "cascade" following an incidental finding that resulted in NO clinically important or intervenable outcome?

Daily
Several times a week
Weekly
Monthly
Several times in the year
Once
Never in the past year

12. A "cascade" may cause harm to the patient involved (e.g., problems with medical bills or dissatisfaction with care). If you have ever experienced a cascade following an incidental finding that caused your patient harm, what harms did the cascade(s) cause? (Choose all that apply)

Physical harm (e.g., procedural complication, adverse drug effect)
Psychological harm (e.g., anxiety, stress)
Treatment burden (e.g., time traveling to and attending appointments)
Disrupted social relationships or status (e.g., stigma related to new diagnosis)
Financial burden (e.g., medical bills, lost wages)
Dissatisfaction with care (e.g., loss of trust in clinician or health system)
Death (Please Describe: ______________________________)
Other (Please Describe: ______________________________)
I have never experienced a cascade that caused my patient harm (Exclusive) Skip to Q. 14

13. In the past year, how often did you experience a "cascade" following an incidental finding that caused your patient harm?

Daily
Several times a week
Weekly
Monthly
Several times in the year
Once
Never in the past year

14. Sometimes "cascades" can cause harm to the physician(s) involved. If you have ever experienced a cascade following an incidental finding that caused you harm, what harm did the cascade(s) cause you? (Choose all that apply)

Anxiety
Frustration
Wasted time and effort
Other (Please Describe: ______________________________)
I have never experienced a cascade that caused me harm (Exclusive) Skip to Instructions after Q.15

15. In the past year, how often did you experience a "cascade" following an incidental finding that caused you harm?

Daily
Several times a week
Weekly
Monthly
Several times in the year
Once
Never in the past year

- If Q.8 is "I have never experienced a cascade that uncovered something clinically important and intervenable" skip to instructions after Q. 16
- Otherwise, ask Q. 16

16. Did your experience of a "cascade" following an incidental finding that uncovered something clinically important and intervenable make you more likely to order the same initial test in the future?

Yes
No
Not sure

- If Q. 10 is "yes", ask Q.17
- Otherwise, skip to instructions after Q. 17

17. Did your experience of a "cascade" following an incidental finding that resulted in NO clinically important or intervenable outcome make you less likely to order the same initial test in the future?

Yes
No
Not sure

- If Q. 12 AND Q.14 is "I have never experienced a cascade that caused my patient or the physician involved any harm, Skip to Q.19
- Otherwise, ask Q. 18

18. Did your experience of a "cascade" following an incidental finding that caused harm make you less likely to order the same initial test in the future?

Yes
No
Not sure

19. Think back to the last time you experienced any "cascade" from an incidental finding for your patient. Who ordered the initial test that showed the incidental finding?

You
A generalist physician colleague (including primary care, emergency, or hospital medicine)
A generalist nurse practitioner or physician assistant colleague (including primary care, emergency, or hospital medicine)
A specialist

20. Thinking back to this last time, what was the initial test that showed the incidental finding? (Randomize list)

Blood test, not microbiology or pathology
Cardiac test (e.g., electrocardiogram)
Imaging test
Microbiology or pathology test (e.g., stool culture, Papanicolaou smear)
Procedure (e.g., colonoscopy)
Urine or stool test, not microbiology or pathology
Other (Please Describe: ____)

21. Thinking back to this last time, in what context was this initial test ordered? (Randomize List)

Research study — Skip to Q. 24
Diagnostic evaluation for a new problem
Screening/preventive care
Testing for an ongoing chronic condition
Testing for a prior condition (e.g., post-cancer surveillance)
Other (Please Describe: ____)

22. Thinking back to this last time, where was the initial test ordered?

Emergency Department
Inpatient
Outpatient clinic
Other (Please Describe: ____)

23. Thinking back to this last time, in your opinion, was this initial test clinically appropriate?

Yes
No
Not sure

24. Thinking back to this last time, what, if anything, concerned you about this incidental finding? (Choose all that apply) (Randomize List)

Possibility of an acute event (e.g., heart attack)
Possibility of a new malignancy
Possibility of an undiagnosed chronic disease (not malignancy, e.g. chronic kidney disease, uterine fibroids)
Other (Please Describe: ____)
I was not concerned (Exclusive)

25. Thinking back to this last time, to your knowledge, was a clinical guideline or algorithm available on how to address this incidental finding?

Yes and it was followed
Yes but it was not followed
No
Not sure

26. Thinking back to this last time, what, if anything, led you to pursue additional evaluation? (Choose all that apply) (Randomize list with "other" and "Did not pursue additional evaluation" at end)

Another doctor advised it
I was concerned about being sued if I missed something important
I was following the norms of my practice or medical community
Time constraints
The incidental finding seemed clinically important
The patient asked for it
Other (Please Describe: ____)
I did not pursue additional evaluation (Exclusive)

27. What did this "cascade" uncover that was <u>clinically important and intervenable</u>? (Choose all that apply) (Randomize list)

Acute event (e.g., heart attack)
Malignancy
Detection of a chronic disease or condition (e.g., chronic kidney disease, uterine fibroids)
Other (Please Describe: ____)
It resulted in <u>NO clinically important or intervenable outcome</u> (Exclusive)

28. Thinking back to this last time, what harm(s) did the "cascade" cause the patient? (Choose all that apply) (Randomize with "other" and "no harm" at end)

Physical harm (e.g., procedural complication, adverse drug effect)
Psychological harm (e.g., anxiety, stress)
Treatment burden (e.g., time traveling to and attending appointments)
Disrupted social relationships or status (e.g., stigma related to new diagnosis)
Financial burden (e.g., medical bills, lost wages)
Dissatisfaction with care (e.g., loss of trust in clinician)
Death (Please Describe: ____)
Other (Please Describe: ____)
The cascade did not cause the patient harm (Exclusive)

29. Thinking back to this last time, what harm(s) did the "cascade" cause you? (Choose all that apply)

Anxiety
Frustration
Wasted time and effort
Other (Please Describe: ____)
The cascade did not cause me harm (Exclusive)

30. Thinking back to this last time, how did you attempt to shorten or lessen the impact of the "cascade"? (Choose all that apply) (Randomize list with "other" and "did not shorten or lessen" at end)

Discussed with a generalist colleague
Discussed with a specialist colleague
Discussed with patient
Read guidelines
Read educational reference (e.g. UpToDate)
Read primary literature
Other (Please Describe: ____)
I did not attempt to shorten or lessen the impact (Exclusive)

31. Thinking back to this last time, how did you feel at the end of the "cascade"? (Choose all that apply) (Randomize)

Annoyed
Frustrated
Helpless
Regretful
Relieved
Other (Please Describe: _____________________________________)
None of the above (Exclusive)

32. In general, what do you think might help limit the negative impact of "cascades" following incidental findings? (Choose all that apply) (Randomize)

Accessible guidelines on how to manage incidental findings
Clinician education on managing incidental findings during training or continuing medical education
Evidence-based recommendations for next steps on radiology and laboratory result reports
Malpractice reform
Patient and clinician education on potential harms from unnecessary medical care
Patient cost-sharing (i.e., insurance plan requires patient to pay a portion of medical costs out-of-pocket)
Shared decision making tools to aid conversations with patients
Value-based payment models (e.g., Accountable Care Organizations)
Other (Please Describe: ____)

33. How often have you experienced a "cascade" following an incidental finding as a patient?

Never
Once in my lifetime
More than once in my lifetime

34. How often has any friend or relative of yours experienced a "cascade" following an incidental finding as a patient?

Never
Once in my lifetime
More than once in my lifetime

35. How much do you agree or disagree with each of the following statements?

	Strongly disagree	Moderately disagree	Slightly disagree	Slightly agree	Moderately agree	Strongly agree
Trying to contain costs is the responsibility of every physician.						
There is currently too much emphasis on costs of tests and procedures.						
Doctors need to take a more prominent role in limiting use of unnecessary tests.						
Doctors are too busy to worry about the costs of tests and procedures.						
The cost of a test or medication is only important if the patient has to pay for it out-of-pocket.						
It is unfair to ask physicians to be cost-conscious and still keep the welfare of their patients foremost in their minds.						
I find the uncertainty involved in patient care disconcerting						

36. Which of the following best describes your main outpatient practice site; i.e., the setting in which you provide most of your patient care services?

Solo practice
Small group private practice (< 4 providers) NOT affiliated with an academic medical center
Large group private practice (5+ providers) NOT affiliated with an academic medical center
Academic medical center-based practice
Staff model HMO
Community clinic or community health center (serving low income areas)
Public or government-based practice (e.g., county, state, federal)

Other (Please Describe: ______________________________________)

37. How would you describe your main outpatient practice site?

Urban
Rural
Suburban

38. What is the zip code at your main outpatient practice site?

____

39. Has a medical malpractice lawsuit ever been filed against you?

Yes, once
Yes, more than once
No Skip to Q. 41

40. Has a medical malpractice lawsuit been filed against you related to missed follow-up of an incidental finding?

Yes
No

41. Approximately what percentage of your professional time is spent in direct patient care?

<25%
25-49%
50-74%
75%+

42. Which of the following best describes you?

I am a US medical graduate
I am a foreign medical graduate

43. Are you of Hispanic, Latino or Spanish origin?

Yes
No
Prefer not to answer

44. With which racial group do you identify yourself?

White
Black or African American

Asian or Pacific Islander
Native American or Alaskan native
Of mixed racial background
Some other race
Prefer not to answer

eAppendix 2. Response Rate Calculation

We used the AAPOR response rate 3 definition which takes into account the proportion of cases of unknown eligibility that may in fact be eligible ("e").[1]

$$e = \frac{eligible\ respondents}{total\ respondents} = \frac{376}{443} = 84.9\%$$

$$RR3 = \frac{eligible\ respondents}{eligible\ respondents + e(nonrespondents)} = \frac{376}{376 + .849(548)} = 44.7\%$$

eTable1. Sample Weights

**Weights**	≤39 years	40-55 years	≥56 years
Resident	1.61	3.60	-
Fellow	0.95	2.00	-
Attending	0.93	0.81	0.69

eTable 2. Selected Survey Responses Stratified by Attending vs Trainee Physician Status

Question		Attending Physicians (N=261)	Trainee Physicians (N=115)
Ever experienced an incidental finding for your patient that led to a cascade, % (95% CI)		99.7 (97.6, 100.0)	99.1 (93.7, 99.9)
Ever experienced a cascade following an incidental finding as a patient, % (95% CI)		39.3 (33.5, 45.4)	39.4 (30.7, 48.8)
Friend or relative ever experienced a cascade following an incidental finding as a patient, % (95% CI)		59.4 (49.2, 70.9)	48.1 (34.3, 65.5)
Cost-consciousness scale,[a] mean (95% CI)		26.1 (25.4, 26.7)	23.6 (22.6, 24.6)
Discomfort with uncertainty scale,[b] mean (95% CI)		3.8 (3.6, 3.9)	3.8 (3.6, 4.1)
Medical malpractice lawsuit ever filed against you, % (95% CI)		36.5 (30.9, 42.5)	0.54 (0, 3.8)
Reason to pursue follow-up of incidental finding during most recent cascade, % (95% CI)	Another doctor advised it	18.9 (14.6, 24.2)	19.8 (13.4, 28.2)
	I was concerned about being sued if I missed something important	34.3 (28.7, 40.4)	25.3 (18.1, 34.3)
	I was following the norms of my practice or medical community	43.1 (37.1, 49.3)	41.1 (32.1, 50.6)
	Time constraints	4.1 (2.2, 7.4)	6.1 (2.9, 12.4)
	The incidental finding seemed clinically important	52.6 (46.4, 58.6)	62.1 (52.7, 70.7)
	The patient asked for it	23.6 (18.8, 29.3)	14.6 (9.2, 22.4)
Attempts to shorten/lessen impact of most recent cascade, % (95% CI)	Discussed with a generalist colleague	9.9 (6.7, 14.2)	27.6 (19.8, 37.1)
	Discussed with a specialist colleague	25.7 (20.7, 31.4)	32.1 (24.0, 41.5)
	Discussed with patient	68.0 (62.1, 73.4)	55.8 (46.3, 64.9)
	Read guidelines	30.5 (25.1, 36.5)	49.2 (39.8, 58.6)
	Read educational reference (eg, UpToDate)	41.1 (35.2, 47.3)	52.9 (43.4, 62.1)
	Read primary literature	10.3 (7.1, 14.7)	24.6 (17.2, 33.9)

Values are weighted percentages. [a]The discomfort with uncertainty scale ranged from 1-6, with 6 signifying the greatest discomfort. [b]To create the cost consciousness scale, we reversed items with negative wording to ensure that a higher score meant greater cost-consciousness, then calculated summary scores by summing the responses (6-36, 36 denoted most discomfort).

eFigure. Reported Events and Outcomes of Cascades Following an Incidental Finding, Stratified by Attending vs Trainee Physician Status

A. Attending Physicians, N = 260

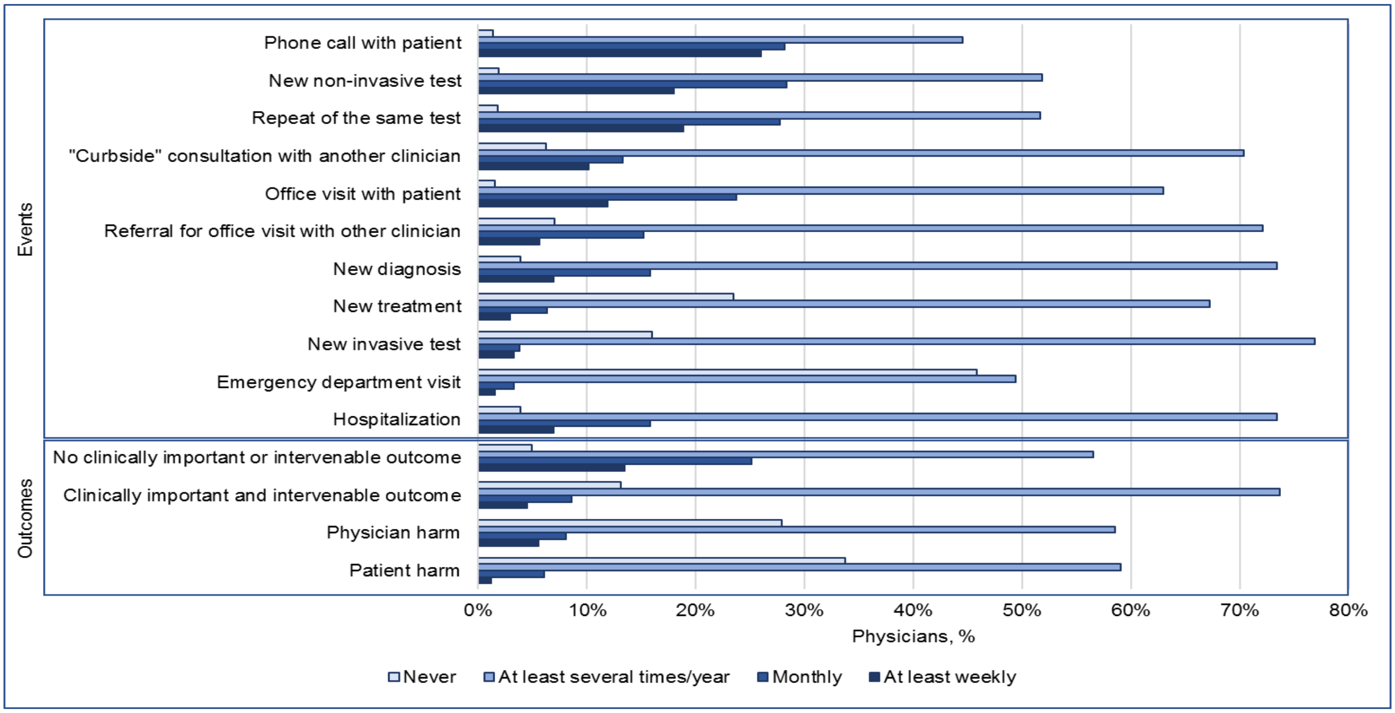

B. Trainee Physicians, N = 114

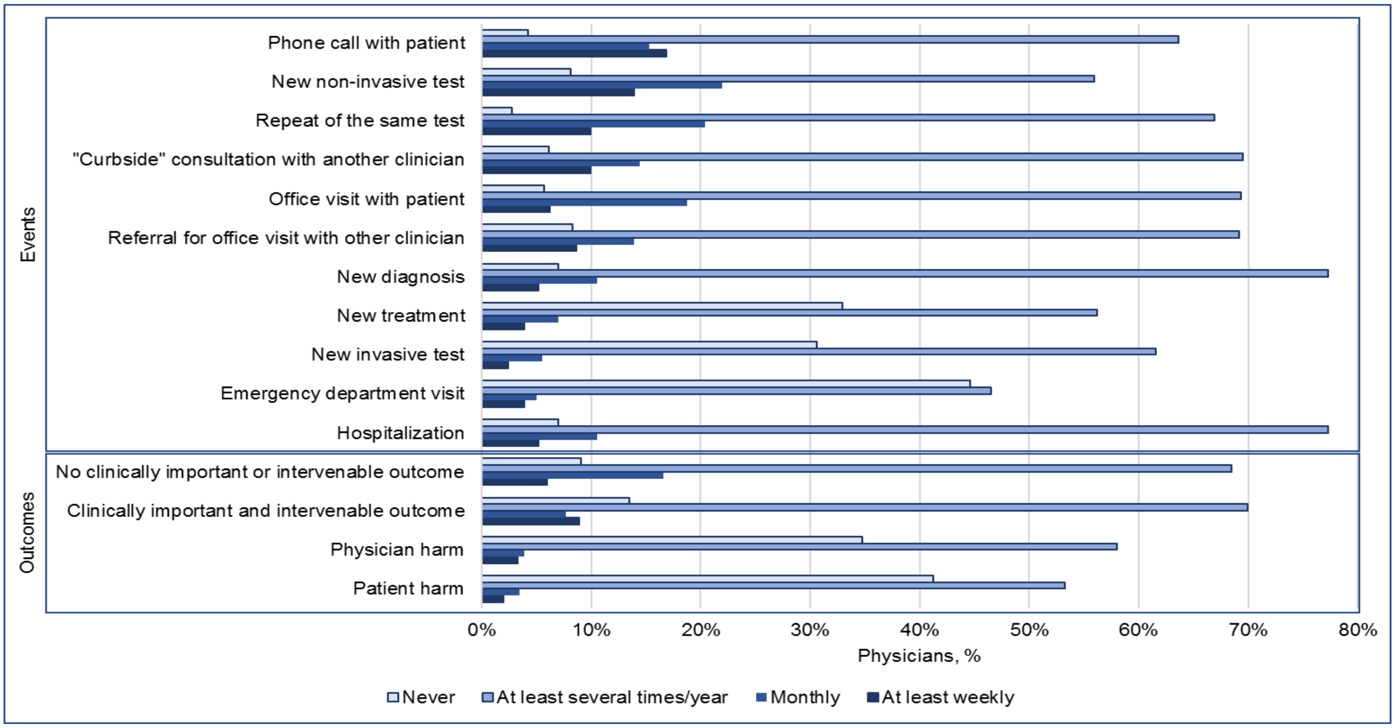

eReference

1. Smith TW. A Revised Review of Methods to Estimate the Status of Cases with Unknown Eligibility. NORC/University of Chicago. www.aapor.org/uploads/. Published 2009. Accessed May 23, 2019.
